# Nutritional Quality of Lunches Served in South East England Hospital Staff Canteens

**DOI:** 10.3390/nu10121843

**Published:** 2018-12-01

**Authors:** Agnieszka Jaworowska, Gabriela Rotaru, Tatiana Christides

**Affiliations:** 1 School of Science, Faculty of Engineering and Science, University of Greenwich, Central Avenue, Chatham ME4 4TB, UK; rg323@gre.ac.uk; 2 Leicester Medical School, College of Life Sciences, George Davies Centre, University of Leicester, University Road, Leicester LE1 7RH, UK; tc243@leicester.ac.uk

**Keywords:** worksite canteen, energy density, nutritional composition, hospital meals

## Abstract

Worksite canteens generally are characterized by obesogenic environments, which offer access to energy-dense foods and sugar-sweetened beverages rather than nutrient-rich food. This study assessed the nutritional quality of hot lunches offered in National Health Service (NHS) hospital staff canteens: 35 side dishes and 112 meals were purchased from 8 NHS hospital staff canteens. The meals were analyzed for portion size, energy, protein, total fat, saturated fatty acids (SFAs), salt, and the sodium to potassium ratio. The vegetarian and meat-based lunch meals served in the hospital staff canteens tended to be high in energy, total fat, saturated fatty acids, and salt: 40%, 59% and 67% of meat meals and 34%, 43%, and 80% of vegetarian meals were assigned the red traffic light label for total fat, salt, and SFAs per portion, respectively. Similar types of meals, but served in different hospitals, varied considerably in their nutritional quality. The consumption of some lunch meals could provide more than 50% of recommended total fat, SFAs, and salt for both men and women and daily energy for women. The majority of analyzed lunch meals were characterized by an unfavorable nutrient profile, and regular consumption of such meals may increase the risk of noncommunicable diseases.

## 1. Introduction

Lifestyle changes that have taken place in many countries worldwide have impacted food consumption patterns, and meals prepared outside the home are currently a regular component of the western diet [1]. The observed dietary changes also affect work-time meal choices, and it has been estimated that approximately 70% of workers consume lunches prepared outside the home, with around 30% of them purchasing their meals from worksite canteens [2,3]. The use of worksite canteens is even higher in the United Kingdom (UK) hospital setting, with 44% of doctors reporting that they purchase their main meals from hospital cafeterias [4]. As the majority of working age people spend a significant part of their waking hours in the workplace [5], the work food environment, and thus foods consumed, may have a significant impact on their overall dietary intake, and thereby on public health [6,7]. Therefore, the workplace may be an ideal setting for health promotion, especially as it provides an opportunity to reach a large number of people. However, despite the potential, worksite canteens generally are characterized by obesogenic environments that offer access to energy-dense foods and sugar-sweetened beverages rather than nutrient-rich food [8,9]. This may underlie research findings that have shown that frequent consumption of food produced outside the home is associated with adverse health outcomes, including an increased risk of hypertension, insulin resistance, type 2 diabetes, cardiovascular disease, and obesity [1,10]. An interesting exception is Finland, where frequent eating in staff canteens has been linked with the increased consumption of fish and vegetables and with overall higher quality of the diet [11]. In addition, men who eat lunch at staff canteens were more likely to be normal weight.

The rising prevalence of obesity nd related comorbidities is one of the major health problems globally [12]. Furthermore, the prevalence of obesity among healthcare workers has been found to be higher than in the general population [13]. In the UK around 61% of nurses and 49% of other healthcare professionals are either obese or overweight [14]. This high prevalence of obesity and associated metabolic complications among people working in the healthcare sector has also been observed in other countries [13,15,16]. Beyond the health risks of being overweight or obes for healthcare staff themselves, this may actually have clinical implications for patient care, as it has been found that lifestyle advice on diet and exercise given by an overweight healthcare professional may be less convincing to patients [13]. Therefore, the workplace food available within healthcare settings may be an especially important public health issue, as the possibility of influencing the dietary intake of health professionals may result in health benefits not only for them, but also for wider society [17].

In recognition of the above, in 2014 the UK Department of Health (DoH) introduced mandatory food standards for UK National Health Service (NHS) hospitals [18] recommending that “hospital canteens must promote healthy diets for staff and visitors and the food offered will need to comply with government recommendations on salt, saturated fats and sugar” [19]. Considering the high prevalence of obesity and its comorbidities among healthcare workers, the high use of worksite canteens in hospitals, and a lack of data regarding the nutritional quality of meals served in hospital staff canteens, there is a need to assess nutritional composition of these meals. Therefore, this study aimed to assess the nutritional quality, including energy, proteins, carbohydrates, total fat, saturated fatty acids (SFAs), fiber, and salt content of hot lunches offered in a selection of NHS hospital staff canteens.

## 2. Materials and Methods

### 2.1. Meals Selection and Collection

All meals were anonymously purchased from eight purposively selected NHS hospitals across South East England. Hospital sites were selected to represent a variety of demographics, including rural and urban sites and differing socioeconomic status [20]. All hospitals were in the secondary healthcare setting. The collection of meals took place between May 2016 and June 2016. Before the meal collection started, detailed analysis of the offered menus was performed to select the most common lunch meals (vegetarian and meat) served across the hospitals. All selected meals were served hot, were freshly prepared on the hospital site, and were purchased once from each selected hospital unless the meal size was too small for nutritional analysis, in which case an additional sample was collected. In total, 25 side dishes and 112 meals (73 meat and 39 vegetarian meals) were purchased from 8 NHS hospitals, with a minimum of 8 meat and 5 vegetarian meals from each hospital. The serving size of each meal was determined by the canteen staff on an “as served” basis without any input from investigators, and thus represented what a customer using the canteen would receive.

### 2.2. Meals Analysis

All samples were weighed on the day of collection, homogenized, and stored at −80 °C until analysis. All sampled meals were analyzed for nutritional composition in July 2016 by Premier Analytical Services (High Wycombe, UK) UKAS (United Kingdom Accreditation Service), an accredited food analysis laboratory. The meals were analyzed for energy (kJ), protein (g), total fat (g), saturated fatty acids (g), carbohydrates (g), fiber (g), and sodium (g) content. Protein content was determined by the Dumas combustion method: Briefly, the samples were decomposed through oxidative combustion to produce oxides of nitrogen. Next, total nitrogen was measured, and crude protein was calculated using the standard conversion factor [21]. Total fat was determined by nuclear magnetic resonance after the samples were dried [22]. Saturated fats were determined based on methyl ester derivatives using gas chromatography. The absolute amounts of saturated fats were calculated by normalization of total fatty acids and the use of a 0.956 conversion factor for nonfatty acid material present in the fat [23]. Total carbohydrates were calculated by difference from sample weight of the other constituents in the food (100 g − (fat + proteins + ash + moisture + fiber)). Dietary fiber was determined using the official method of the Association of Official Analytical Chemists [24]. Energy levels were calculated according to EU (European Union) regulation 1169/2011 on food labeling by taking into consideration the Atwater energy equivalents [25]. 

### 2.3. Statistical Analysis

The data were analyzed using SPSS version 17.0 (SPSS Inc., Chicago, IL, USA), and *p* < 0.05 was considered statistically significant. All variables were checked for normality of distribution using histograms and the Shapiro–Wilk test. All results are presented as medians with interquartile range (25th and 75th percentiles) per 100 g and per portion. Differences in nutritional content between meat and vegetarian meal categories and between different types of meals within either the meat or vegetarian category (the most common meat and vegetarian meal types served across 8 hospitals were selected) were analyzed using the Kruskal–Wallis test and the Mann–Whitney U test, respectively. The nutrient content of meals was compared to the current UK guidelines for planning nutritionally balanced meals for adults 19–50 years old [26]. According to these recommendations, lunch should provide 30% of estimated average requirements (EARs) for energy [27], no more than 30% of dietary reference values (DRVs) for fat and SFAs [28], no more than 30% of a target salt intake of 6 g/day [29], and at least 30% of the recommended fiber intake of 30 g/day [30]. In addition, meals were also classified according to the UK Food Standards Agency (FSA) “traffic light system”, which is a color-coded front-of-pack labeling scheme used in the UK to help consumers make healthier dietary choices [31]. Red indicates high, amber indicates medium, and green indicates low content of total fat, SFAs, sugar, or salt.

## 3. Results

The median energy and nutrient content per 100 g and per portion in meat and vegetarian hospital lunches is presented in Table 1 and Table 2. On average the meat-based meals were characterized by a significantly higher energy density (174 *v* 139 kcal/100 g; *p* < 0.05) when compared to the vegetarian meals. However, the energy content per portion was similar for both types of meals. The meat-based meals were also higher in salt (0.61 *v* 0.49 g; *p* < 0.05) per 100g and in protein both per 100g (9.8 *v* 4.8 g; *p* < 0.05) and per portion (32.9 *v* 19.0 g; *p* < 0.05), and tended to be higher in fat and fiber and lower in SFAs when compared to the vegetarian lunches.

In addition, significant differences in the nutritional composition between different types of meals but from the same category were observed (Table 1 and Table 2). Among meat-based meals, cottage pie provided 100% less energy than a portion of sausage with chips (435 *v* 1020 kcal/per portion; *p* < 0.05). Similarly a portion of chicken curry with rice contained approximately six times less total fat than a portion of sausage with chips or fish with chips (Table 2). A similar trend was observed in the vegetarian meal category: A portion of pasta with tomato sauce contained half the amount of total fat and salt in comparison to vegetarian lasagna or macaroni and cheese (Table 2). 

**Figure 1:**
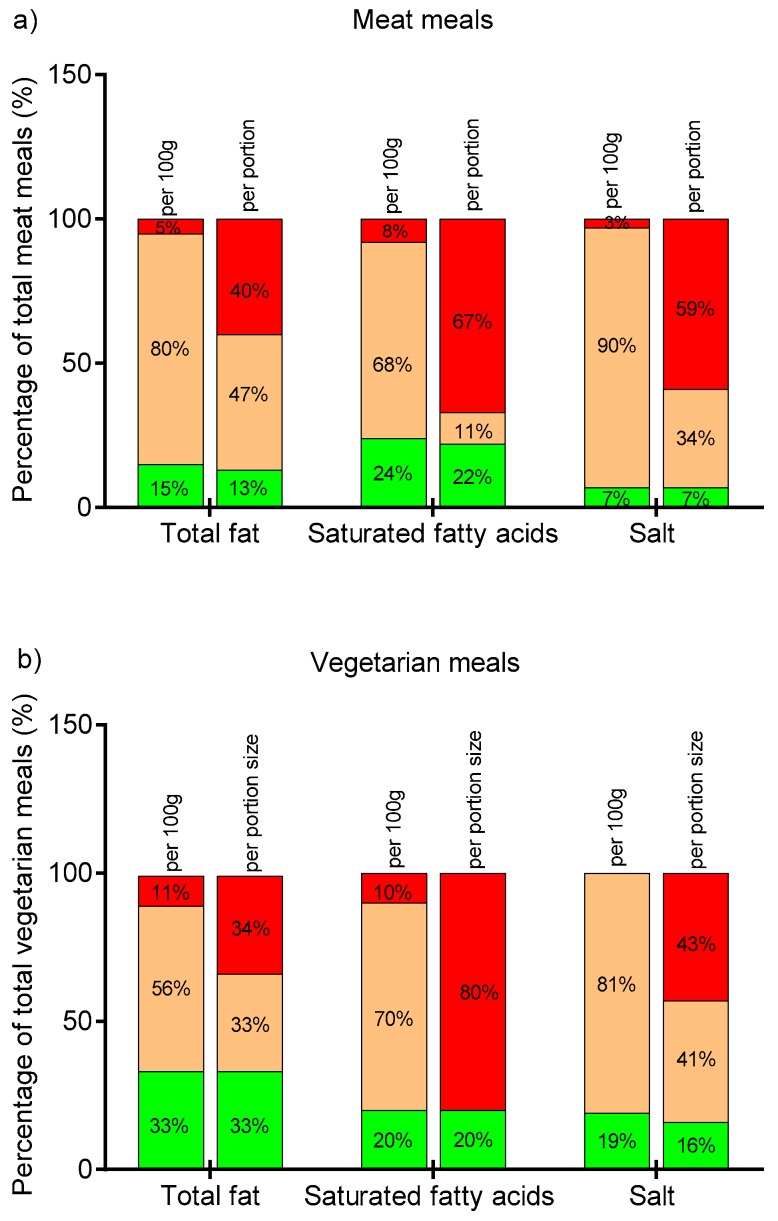
Traffic light distribution of meals per 100g and per portion based on the United Kingdom Food Standards Agency guidelines. (**a**) Meat category: total fat (*n* = 73), saturated fatty acids (SFAs) (*n* = 39), salt (*n* = 73); (**b**) vegetarian category: total fat (*n* = 39), SFAs (*n* = 10), salt (*n* = 39). Red indicates high, amber is medium, and green is low content of total fat, SFAs, and salt.

The nutritional content of hospital lunches was also compared to the Food Standard Agency traffic light labeling scheme (Figure 1). Whereas the majority of meals fell into the amber category when looking at total fat, SFAs, and salt per 100 g, the situation changed dramatically when analyzing the same nutritional profile, but per portion. The biggest difference was observed for SFAs and salt content for both meat and vegetarian meals: 8% of meat and 10% of vegetarian meals were classified into the red traffic light category based on SFA content per 100 g, but 67% and 80% of these meals, respectively, were classified as “red” based on the SFA amount per portion. Of note, although meat-based meals are typically considered a primary source of SFAs, in this study vegetarian meals on average had higher SFA levels (Table 1 and Table 2). Similarly, 69% of meat and 43% of vegetarian meals were in the red category when the salt content per portion was considered. However, none of the vegetarian and only 3% of the meat meals were classified as “red” based on the amount of salt per 100 g. It is worth noting that only a small proportion of lunch meals were categorized as “green”, regardless of the nutrient considered and regardless of the classification of either per portion or per 100 g of the meal.

**Figure 2:**
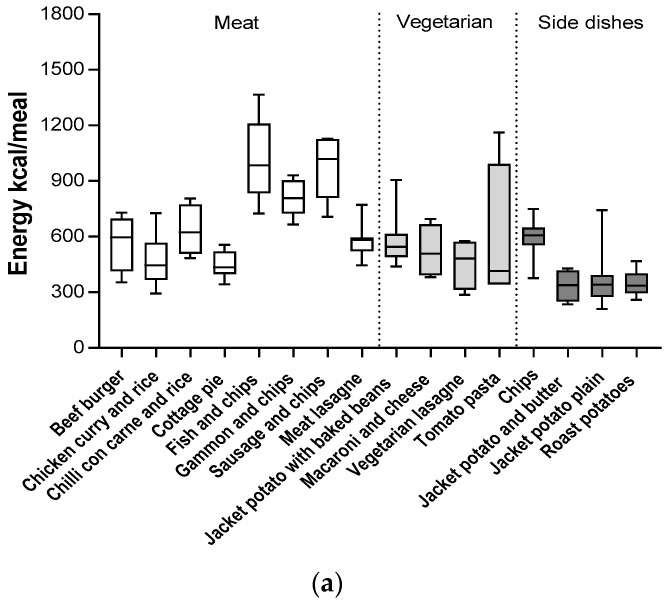

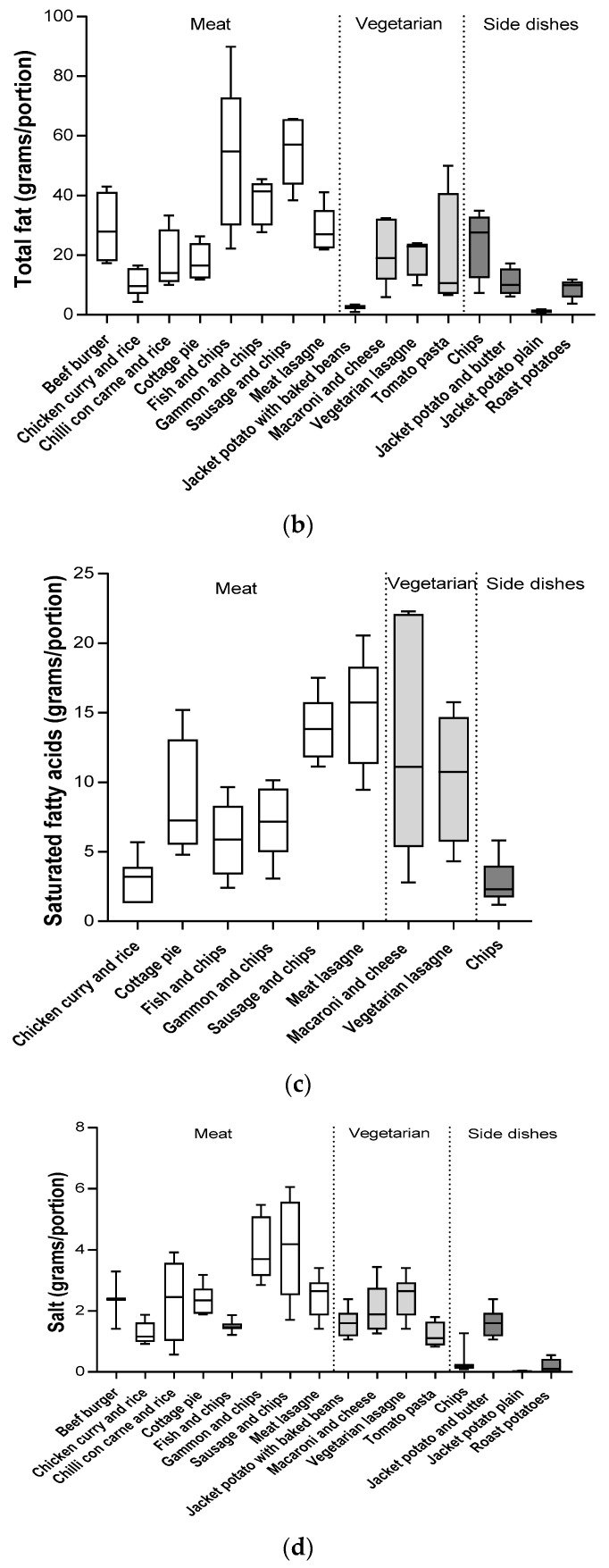
Variation in (**a**) energy, (**b**) total fat, (**c**) SFAs, and (**d**) salt for meat and vegetarian meals groups and for side dishes.

Interestingly, we observed that similar types of meals, but served in different hospitals, varied considerably in nutritional quality (Table 1 and Table 2, Figure 2). For example, 100 g of pasta with tomato sauce could contain between 90–216 kcal of energy and between 1.0–8.0 g of fat depending on the hospital from which it had been purchased (Figure 2). Similarly, the energy densities of fish and chips served in different hospitals ranged from 207 to 302 kcal/100 g, fat content per 100 g varied from 5.1 to 18.7 g, salt varied from 0.19 to 0.55 g, and SFAs varied from 0.55 to 2.4 g. In addition to the variation of the nutrient content per 100 g, there was an even greater variability of the nutritional profile per portion. This was influenced by the inconsistency of portion sizes of similar types of meals across different hospitals (Table 2). Spearman’s correlation analysis revealed that not only energy density, but also portion size, significantly impacted the energy content of the analyzed meals. A similar effect was observed for SFAs and salt, although the nutrient content of the meal was more influenced by nutrient density rather than portion size (Table 3).

Table 4 shows the nutritional composition of the hospital lunches per portion in comparison to the UK DRVs. The vegetarian meals provided 15–21% and 20–26% of the EAR for energy and 3–23% and 3–29% of the DRV of total fat for men and women, respectively. However, these meals provided more than the recommended 30% of the DRV of SFAs for both men and women, and some meals also contained more than 30% of the DRV of salt. The meat-based meals varied from 17 to 39% and from 21 to 49% of the EAR for energy for men and women, respectively. Around 50% of the meat-based meals provided more than the recommended 30% of daily intake of energy and fat. In addition, salt content of six out of eight analyzed meat meal types ranged from 31% to 63% of the recommended target of 6 g/day (Table 4). Surprisingly, the meat meals tended to provide more fiber (22% DRV) than the vegetarian lunches (16% DRV). In addition, meat-based meals were characterized by very high protein content that ranged from 54% to 73% of Recommended Nutrient Intake (RNI) for men and from 65 to 85% of RNI for women.

Importantly, there were no statistically significant differences in the nutritional quality of meals served in hospital staff canteens in relation to their location (urban vs. rural) or socioeconomic status of the area.

## 4. Discussion

The vegetarian and meat-based lunch meals served in NHS hospital canteens tended to be high in energy, total fat, SFAs, and salt, and low in fiber: 40%, 59%, and 67% of meat meals and 34%, 43%, and 80% of vegetarian meals were assigned the red traffic light label for total fat, salt, and SFAs per portion. It should also be noted that meals were analyzed as offered, not as ordered by consumers, which means that some of them could be purchased with additional side dishes. For example, burgers are often consumed with chips. In addition, chips were analyzed without salt added at the table, and no information on drinks consumed with meals was collected. Therefore, it could be predicted that actual intake of nutrients may be higher than estimated based on nutritional analysis of sampled foods alone.

To the best of our knowledge, this is the first study to assess the nutritional profile of lunch meals served in hospital canteens. There has been one previous study that assessed salt content only, reporting that an average lunch containing soup and a nonvegetarian hot dish, served in university and non-university hospital staff canteens in the Netherlands, provided 7.4 g (±0.5 g) and 7.0 g (±0.3 g) of salt, respectively [32]. The average salt content in the meals served in the UK hospital staff canteens was lower and ranged from 0.99 to 2.15 g per portion in the vegetarian meals and from 0.92 to 3.70 g per portion in the meat-based meals. However, the results of our study are comparable to the nutritional quality of UK ready meals, which have also been reported to be high in total fat, SFAs, and salt [33]. Furthermore, the energy density of the analyzed meat-based meals ranged from 131 to 231 kcal/100 g and was similar to the reported energy density of UK takeaway meals (102 and 302 kcal/100 g) [34]. However, the total energy content per meal was considerably lower in lunches served in hospital staff canteens than in takeaway meals, which was related to the smaller portion sizes. The meals offered in UK hospital canteens had higher energy densities compared to lunch meals eaten at Danish (143 kcal/100 g) [35] and Belgian (129 kcal/100 g) [36] worksite canteens. UK canteen meals provided 40% more energy per 100g than is currently recommended in diets intended to prevent obesity [37]. It is possible that the lack of implementation of nutritional standards in UK hospital staff canteens may be related to funding issues, as the NHS is under severe financial pressure and patient care is an identified priority [38].

Considering the frequent use of staff canteens [2,3,4] and the high prevalence of obesity and associated metabolic complications among health professionals [13,14,15,16], the nutritional quality of food served in hospital staff canteens is an important issue. Previous studies have often reported that frequent consumption of food produced outside the home, which is characterized by poor nutritional quality, is associated with adverse health outcomes [1]. Similarly, the Oslo Health Study found a significantly increased likelihood of being obese (BMI ≥ 30 kg/m^2^) with frequent eating in staff canteens [10]. Our study showed that the consumption of some lunch meals could provide greater than 50% of the recommended daily intake of total fat, SFAs, and salt for both men and women, and more than 50% of the recommended daily energy for women. Therefore, regular consumption of such meals may increase the risk of obesity, hypertension, and other metabolic conditions among hospital staff.

One of the NHS roles is to promote healthy living to encourage people to improve their health and well-being [39], and thus it would be helpful if hospital settings and healthcare professionals were “role models” of a healthy lifestyle. The World Health Organization Ottawa Charter for Health Promotion suggests “Health Promoting Hospitals” as a strategy to promote health in the community, with hospitals acting as an advocate and a “change agent” [40]. Hospital canteens serving nutritious meals could advocate healthy eating for all, and so promote health in their communities as well as their healthcare staff. Nutrition interventions within the healthcare sector could thus potentially bring health benefits not only to its workforce but also to wider society.

Interestingly, it was observed that similar type of meals, but served in different hospitals, varied considerably in their nutritional quality. This nutritional variability of similar types of meals between different hospitals suggests that recipe reformulation should be explored as a possible strategy to improve the nutritional quality of meals served in hospital staff canteens. A cross-sectional comparison study investigating dietary intakes of adults working in two public sector hospitals in Ireland found that catering initiatives based on menu modification could promote healthy eating and reduce dietary intakes of energy, total fat, SFAs, sugar, and salt [41]. Similarly, a nutritional program implemented in cafeterias on hospital campuses in Ottawa (Canada) involving the provision of nutrition information on menus and an improvement in the nutritional quality of meals offered resulted in lower intakes of energy, sodium, total fat, and SFAs [42]. This clearly suggests that the type of food that is available at a workplace may influence dietary intake. This may have an even greater impact on the diet of healthcare professionals, who, due to irregular breaks and the location of hospitals (i.e., a lack of external food sources near hospitals) often need to rely on hospital canteens as their only work meals provider. 

This study had several strengths. The nutrient content of meals was measured directly by an accredited food laboratory. The nutrient content of food products can be determined using food composition tables, but direct laboratory analysis is the most reliable method [43]. Detailed analysis of each hospital menu was performed to select the most common lunch meals, which allowed more adequate comparisons between hospitals. Hospitals were selected to represent a variety of demographics including rural and urban sites and differing socioeconomic status. A number of limitations need to also be considered. Only one sample of each meal was purchased from each hospital canteen, and therefore our results may not be an adequate representation of selected meals’ nutritional profiles. Moreover, only eight hospitals were included, which represents just a small fraction of all UK NHS hospitals. Furthermore, we observed that hospitals canteens differed not only in meal type and portion size, but also in the provision of nutritional information on the menu. Inclusion of vegetables as a part of the meal also varied. All of these factors could affect staff food choice and therefore dietary intake.

## 5. Conclusions

In conclusion, the majority of analyzed lunch meals were characterized by an unfavorable nutrient profile, and regular consumption of such meals may increase the risk of obesity, hypertension, and other metabolic conditions among canteen users. The results of this study should encourage the development of strategies to improve worksite nutrition environment. Recipe reformulations to improve the nutritional profile of meals offered at worksite canteens is warranted. Considering the high frequency of eating in staff canteens, in future research, there is a need to assess the relationship between the food consumption at staff canteens and overall dietary intake.

## Figures and Tables

**Table 1:** Nutritional composition of meals served in hospital staff canteens per 100 g.

Meal Type *	*n*	Energy (kcal/100 g)	Proteins (g/100 g)	Total Fat (g/100 g)	SFAs (g/100 g)	Carbohydrates (g/100 g)	Fiber (g/100 g)	Salt (g/100 g)
Vegetarian		139 (98–173) ^1^	4.8 (3.7–6.2) ^1^	5.8 (1.7–9.5)	4.0 (2.23–4.38) ^#^	16.6 (14.0–19.1)	1.30 (0.95–2.23) ^#^	0.49 (0.33–0.62) ^1^
Jacket potato with baked beans	8	96 (93–98) ^a^	3.7 (3.5–3.8) ^a^	0.6 (0.5–0.6) ^a,b^	n/d	17.8 (16.4–18.8)	n/d	0.36 (0.30–0.42)
Vegetarian lasagna	4	154 (103–172)	6.8 (4.9–6.2)	6.0 (3.9–8.7)	4.0 (2.0–4.3)	14.8 (11.8–18.9)	2.20 (1.38–2.35)	0.61 (0.57–1.01)
Macaroni and cheese	6	152 (117–159)	6.0 (4.2–6.5)	5.7 (2.8–8.5)	3.7 (2.1–5.0)	14.2 (13.3–21.0)	1.15 (0.70–1.55)	0.55 (0.46–0.62)
Pasta with tomato sauce	4	121 (100–197)	4.6 (3.9–5.5)	3.1 (2.1–7.0)	n/d	18.2 (15.3–24.9)	n/d	0.30 (0.26–0.33)
*p*-value ^I^		0.010	0.013	0.001	>0.05	>0.05	>0.05	>0.05
Meat	73	174 (131–231)	9.8 (7.4–12.8)	7.0 (4.1–12.1)	2.00 (1.35–3.55) ^~^	16.7 (10.5–21.1)	1.70 (1.40–2.35) ^~^	0.61 (0.41–0.87)
Beef burger	4	250 (236–283)	14.3 (12.7–16.1) ^c,d,j,k^	12.9 (9.6–6.3)	n/d	21 (12.9–27.8)	n/d	1.03 (0.98–1.03)
Chicken curry and rice	4	123 (116–143) ^c,d,e^	9.6 (4.9–13.5)	3.3 (1.7–3.8) ^c,d,e^	0.8 (0.4–1.4) ^c,g^	14.6 (13.2–20.4)	1.40 (0.88–1.63)	0.27 (0.24–0.45) ^f,c^
Chili con carne and rice	4	125 (121–157) ^d,e^	8.4 (6.4–9.6) ^j^	3.1 (2.3–6.1) ^d,e^	n/d	17.00 (16.1–17.5)	n/d	0.49 (0.22–0.74)
Cottage pie	6	132 (109–144) ^c,d,e^	11.0 (7.0–12.5)	5.0 (3.7–6.4)	2.0 (1.8–3.8)	9.5 (7.9–14.1) ^c,d,e,f^	1.70 (0.70–2.32)	0.59 (0.52–0.83)
Fish and chips	8	234 (213–273)	7.9 (7.5–8.7) ^f,g^	14.4 (9.4–17.8)	1.5 (1.0–2.1) ^g^	22.8 (20.8–25.5)	2.60 (1.93–3.41)	0.35 (0.28–0.52) ^f,c^
Gammon and chips	5	117 (102–177)	12.2 (10.4–15.5)	8.1 (6.8–10.3)	2.0 (1.4–2.4)	16.7 (14.8–19.2)	1.80 (1.55–2.50)	1.12 (1.03–1.31)
Meat lasagna	7	188 (171–199)	12.3 (7.2–14.7)	9.7 (6.2–11.3)	4.6 (3.5–5.9)	10.5 (10.3–11.8) ^c,e,f,i^	1.70 (1.00–1.90)	0.80 (0.42–0.93)
Sausage and chips	5	241 (216–247)	7.3 (6.6–7.7) ^f,g^	13.2 (12.6–14.3)	3.4 (3.1–3.6)	21.2 (17.8–30.7)	2.50 (1.95–2.85)	1.03 (0.89–1.11)
*p*-value ^I^		<0.001	0.005	<0.001	<0.001	<0.001	0.011	0.004
Side dish	25	136 (98–196)	2.6 (2.0–2.9)	2.9 (0.6–6.0)	0.8 (0.6–1.7)	23.8 (20.5–29.3)	3.45 (3.20–3.85)	0.04 (0.00–0.07)
Chips	8	223 (192–257)	3.2 (2.7–3.4)	10.6 (5.1–12.9)	0.8 (0.6–1.7)	30.1 (27.9–35.8)	3.45 (3.20–3.85)	0.07 (0.05–0.08)
Jacket potato with butter	4	121 (89–134) ^h^	2.0 (1.8–2.2) ^h^	3.6 (2.3–5.7)	n/d	19.4 (14.7–20.5) ^h,l^	n/d	0.07 (0.03–0.07)
Jacket potato plain	8	97 (90–103) ^h^	2.2 (1.8–2.5) ^h^	0.4 (0.2–0.6) ^h^	n/d	20.9 (17.8–23.0) ^h,l^	n/d	0.00 (0.00–0.002) ^h^
Roast potatoes	5	156 (141–171)	2.7 (2.2–2.8) ^h^	4.7 (2.4–5.4)	n/d	25.8 (24.4–30.8)	n/d	0.04 (0.01–0.20)
*p*-value ^I^		<0.001	0.002	<0.001	n/a	<0.001	n/a	0.003

* Data presented as median and interquartile (25th and 75th percentile); *n* = number of meals; SFAs = saturated fatty acids; n/d = no data available; n/a = not applicable; ^#^ 10 samples; ^~^ 39 samples; ^1^ significant difference between vegetarian and meat meal options (Mann–Whitney U test, *p* < 0.05); ^I^ difference within each meal category (Kruskal–Wallis). Significant difference between meal types within the same category (Mann–Whitney U test with Bonferroni adjustment, *p* < 0.05): ^a^ macaroni and cheese, ^b^ vegetarian lasagna, ^c^ sausage and chips, ^d^ fish and chips, ^e^ beef burger, ^f^ gammon and chips, ^g^ lasagna, ^h^ chips, ^i^ chicken curry and rice, ^j^ chili con carne and rice, ^k^ cottage pie, ^l^ roast potatoes. Gammon = traditional British pork steak; chips = thick-cut French fries.

**Table 2:** Nutritional composition of meals served in hospitals staff canteens per portion.

Meal Type *	*n*	Weight (g/portion)	Energy (kcal/portion)	Proteins (g/portion)	Total Fat (g/portion)	SFAs (g/portion)	Carbohydrate (g/portion)	Fiber (g/portion)	Salt (g/portion)
Vegetarian	39	380 (310–485)	526 (434–652)	19.0 (14.7–23.0) ^1^	20.8 (5.9–32.3)	11.01 (5.76–17.32) ^#^	66.9 (37.9–87.1)	4.82 (2.81–7.30) ^#^	1.61 (1.06–2.50)
Jacket potato with baked beans	8	551 (497–634) ^a,b,c^	546 (490–614)	19.1 (11.7–19.7)	2.6 (2.0–3.2) ^a,b^	n/d	102.7 (89.6–112.6) ^b^	n/d	1.38 (0.98–2.43)
Vegetarian lasagna	4	353 (257–410)	489 (314–633)	22.0 (11.7–28.1)	23.4 (13.1–26.1)	10.76 (5.76–14.68)	45.9 (32.9–67.1)	6.35 (3.44–9.16)	2.15 (1.49–4.13) ^c^
Macaroni and cheese	6	368 (325–405)	508 (393–667)	21.5 (15.1–27.1)	19.1 (11.9–32.2)	11.10 (5.37–22.10)	59.0 (42.2–77.7)	3.51 (2.51–5.86)	1.97 (1.27–2.72)
Pasta with tomato sauce	4	349 (334–535)	414 (343–993)	20.7 (13.6–22.7)	10.7 (7.0–40.8)	n/d	70.5 (55.9–115.4)	n/d	0.99 (0.85–1.14)
*p*-value ^I^		0.008	>0.05	>0.05	0.002	>0.05	0.012	>0.05	0.032
Meat	73	354 (281–420)	584 (441–781)	32.9 (24.0–40.5) ^1^	22.2 (14.6–35.3)	7.25 (5.31–12.35) ^~^	56.8 (32.3–85.9)	6.40 (4.43–9.40) ^~^	1.89 (1.29–2.87)
Beef burger	4	242 (167–255) ^d,e,f^	597 (414–697)	24.6 (21.5–41.0)	27.0 (22.4–35.0)	n/d	40.1 (28.5–64.3)	n/d	2.39 (1.42–2.39)
Chicken curry and rice	4	341 (300–455)	444 (367–566) ^d,e^	30.4 (16.3–54.8)	9.6 (7.15–15.5) ^d,e,i^	3.20 (1.35–3.09) ^d,j^	43.3 (39.7–94.3)	4.22 (3.02–6.72)	0.92 (0.89–1.48) ^e,i^
Chili con carne and rice	4	480 (419–514)	624 (507–773)	40.1 (27.0–49.7)	14.1 (11.0–28.5)	n/d	83.1 (70.5–84.6) ^j,k,^	n/d	2.46 (1.03–3.57)
Cottage pie	6	344 (315–390)	435 (398–524) ^d,e^	29.9 (24.9–44.9)	16.5 (12.3–24.6) ^e^	7.25 (5.53–13.20)	32.4 (29.2–46.6)	5.49 (2.64–7.66)	2.01 (1.73–3.17)
Fish and chips	8	416 (358–434)	985 (835–1210)	29.1 (23.8–36.9)	28.0(18.1–41.1)	5.87 (3.40–8.28) ^j^	95.0 (87.6–107.4) ^i,j,k,l^	9.90 (8.28–11.00)	1.51 (0.97–1.56) ^e,i^
Gammon and chips	5	399 (349–406)	808 (725–904)	36.2 (32.4–49.0)	54.8 (30.1–72.8)	7.17 (4.99–9.54)	73.5 (68.8–81.6)	8.68 (6.71–9.38)	3.32 (2.58–4.51)
Meat lasagna	7	342 (261–355) ^f^	583 (521–595)	34.6 (28.2–43.6)	41.4 (30.0–44.1)	15.73 (11.34–18.29)	36.5 (25.6–40.3)	4.45 (3.10–5.81) ^d,e^	1.85 (1.49–2.88)
Sausage and chips	5	452 (395–459)	1020 (809–1126)	29.8 (20.8–34.0)	57.1 (43.8–65.5)	13.82 (11.82–14.68)	90.8 (78.7–94.6) ^j,k,l^	11.03 (8.17–12.54)	3.70 (2.48–5.17)
*p*-value ^I^		<0.001	<0.001	>0.05	<0.001	<0.001	<0.001	<0.001	0.004
Side dish	25	280 (216–333)	375 (324–569)	7.0 (7.0–9.3)	9.9 (1.7–15.5)	2.32 (1.74–4.00)	70.5 (53.2–77.0)	8.58 (7.92–9.67)	0.11 (0.00–0.20)
Chips	8	266 (214–293)	607 (554–649)	8.3 (6.7–9.0)	27.6 (12.4–32.9)	2.32 (1.74–4.00)	76.7 (72.8–99.9)	8.58 (7.92–9.67)	0.19 (0.14–0.20)
Jacket potato with butter	4	286 (243–355)	338 (249–418)	5.9 (4.4–7.5)	10.0 (7.0–15.5)	n/d	49.4 (41.0–70.7)	n/d	0.22 (0.11–0.22)
Jacket potato plain	8	334 (267–438)	341 (275–393) ^h^	8.1 (5.1–9.0)	1.2 (0.7–1.7) ^h^	n/d	71.3 (61.0–76.0)	n/d	0.00 (0.00–0.01) ^h^
Roast potatoes	5	215 (197–249) ^g^	335 (294–402) ^h^	5.5 (4.8–6.6)	9.9 (5.9–11.0)	n/d	54.8 (51.0–74.0)	n/d	0.14 (0.02–0.42)
*p*-value ^I^		0.030	0.011	>0.05	<0.003	n/a	>0.05	n/a	0.003

* Data presented as median and interquartile (25th and 75th percentile); *n* = number of meals, n/d = no data available, n/a = not applicable; ^#^ 10 samples; ^~^ 39 samples; ^1^ significant difference between vegetarian and meat meal options (Mann–Whitney U test, *p* < 0.05); ^I^ difference within each meal category (Kruskal–Wallis). Significant difference between meal types within the same category (Mann–Whitney U test with Bonferroni adjustment, *p* < 0.05): ᵃ vegetarian lasagna, ᵇ macaroni and cheese, ^c^ pasta with tomato sauce, ᵈ fish and chips, ᵉ sausage and chips, ^f^ chili con carne and rice, ᵍ jacket potato plain, ^h^ chips, ^i^ gammon and chips, ^j^ meat lasagna, ^k^ cottage pie, ^l^ beef burger. Gammon = traditional British pork steak; chips = thick-cut French fries.

**Table 3:** Correlation between nutrient density, portion size, and nutrient content of the meals.

	Portion Size *r_s_*	Nutrient Density (nutrient per 100 g) *r_s_*
Energy/meal	0.487 *	0.506 *
Total fat/meal	0.116	0.882 *
SFAs/meal	0.109	0.922 *
Salt/meal	0.228 *	0.777 *

*r* = Spearman’s correlation coefficient; * statistically significant, *p* < 0.05.

**Table 4:** Energy and macronutrients profile of the meals as percentage of UK daily reference values for healthy adults (19–50 years old).

Meal Type *	*n*	Energy (% EAR) Men	Energy (% EAR) Women	Proteins (% RNI) Men	Proteins (% RNI) Women	Total Fat (% DRV) Men	Total Fat (% DRV) Women	SFA (% DRV) Men	SFA (% DRV) Women	Carbohydrate (% DRV) Men	Carbohydrate (% DRV) Women	Salt % RNI	Fiber % DRV
Vegetarian	39	20 (16–25)	25 (21–31)	34 (27–42) ^1^	42 (33–51) ^1^	21 (6–32)	26 (7–40)	34 (18–54) ^#^	43 (23–68) ^#^	20 (12–27)	27 (15–36)	27 (18–42)	16 (9–21) ^#^
Jacket potato with baked beans	8	21 (19–24)	26 (24–30)	35 (32–36)	42 (39–44)	3 (2–3) ^a,b^	3 (2–4) ^a,b^	n/d	n/d	31 (27–34) ^a,b^	42 (37–46) ^a,b^	23 (16–41)	n/d
Vegetarian lasagna	4	15 (12–20)	24 (15–30)	40 (21–51)	49 (26–62)	23 (13–26)	29 (16–32)	34 (18–46)	42 (23–58)	14 (10–21)	19 (13–27)	36 (25–69)	21 (11–31)
Macaroni and cheese	6	20 (15–26)	24 (19–32)	39 (27–49)	48 (34–60)	19 (12–32)	24 (15–40)	35 (17–69)	44 (21–87)	18 (13–24)	24 (17–32)	33 (21–45)	12 (8–20)
Pasta with tomato sauce	4	16 (14–38)	20 (17–48)	38 (25–41)	46 (30–50)	11 (7–40)	13 (9–50)	n/d	n/d	22 (17–35)	29 (23–47)	16 (14–19)	n/d
*p*-value ^I^		>0.05	>0.05	>0.05	>0.05	0.002	0.002	>0.05	>0.05	0.012	0.012	0.097	>0.05
Meat	73	22 (17–30)	28 (21–37)	60 (44–74)	73 (53–90)	22 (14–35)	27 (18–43)	23 (16–39) ^~^	29 (20–49) ^~^	17 (10–26)	23 (13–35)	31 (22–49)	22 (15–32) ^~^
Beef burger	4	23 (16–27)	29 (20–34)	63 (39–75)	77 (48–91)	29 (20–34)	35 (22–51)	n/d	n/d	12 (9–19) ^c,d,e^	17 (12–26) ^c,d,e^	40 (24–40)	n/d
Chicken curry and rice	4	17 (14–22) ^c,d^	21 (18–27) ^c,d^	55 (30–100)	67 (36–122)	21 (18–27) ^c,d^	12 (9–20) ^c,d,e^	10 (4–13) ^d,f^	13 (5–15) ^d,f^	13 (12–29)	18 (16–36)	15 (15–25) ^d,e^	14 (10–22)
Chili con carne and rice	4	24 (20–30)	30 (24–37)	73 (49–90)	89 (60–110)	30 (24–37)	17 (14–35)	n/d	n/d	25 (22–26)	34 (29–35)	41 (17–59)	n/d
Cottage pie	6	17 (15–20) ^c,d^	21 (19–25) ^c,d^	54 (45–82)	66 (55–100)	21 (19–25) ^c,d^	20 (15–30) ^d^	23 (17–41)	29 (22–52)	10 (9–14) ^c,d,e,h^	13 (12–19) ^c,d,e,h^	33 (29–53)	18 (9–26)
Fish and chips	8	38 (32–46)	47 (40–58)	53 (43–67)	65 (53–82)	47 (40–58)	68 (37–90)	18 (11–26) ^f^	23 (13–33) ^f^	29 (27–33)	39 (36–44)	25 (16–26) ^d,e^	33 (28–37)
Gammon and chips	5	31 (28–35)	39 (35–44)	66 (59–89)	80 (72–109)	39 (35–44)	51 (37–54)	22 (16–30)	28 (20–38)	22 (21–25)	30 (28–33)	55 (43–75)	29 (22–31)
Meat lasagna	7	22 (20–23)	28 (25–29)	63 (51–79)	78 (63–97)	28 (25–29)	33 (28–43)	49 (35–57)	62 (45–72)	11 (8–12) ^c,d,e,h^	15 (10–16) ^c,d,e,h^	31 (25–48)	15 (10–19)
Sausage and chips	5	39 (31–43)	49 (40–54)	54 (38–62)	66 (46–76)	49 (40–54)	70 (54–81)	43 (37–49)	54 (47–62)	28 (24–29)	37 (32–39)	62 (4186)	37 (27–42)
*p*-value ^I^		<0.001	<0.001	>0.05	>0.05	<0.001	<0.001	<0.001	<0.001	<0.001	<0.001	<0.001	>0.05
Side dish	25	14 (12–22)	18 (16–27)	13 (10–16)	16 (12–19)	10 (2–15)	12 (2–19)	7 (5–12)	9 (7–16)	29 (16–24)	29 (12–31)	2 (0–3)	29 (26–32)
Chips	8	23 (21–25)	29 (27–31)	15(12–16)	18 (15–20)	27 (12–33)	34 (15–41)	7 (5–12)	9 (7–16)	23 (12–31)	31 (30–41)	3 (2–3)	29 (26–32)
Jacket potato with butter	4	13 (10–16) ^g^	16 (12–20) ^g^	11 (8–14)	13 (10–17)	10 (7–15)	12 (9–19)	n/d	n/d	15 (13–22)	20 (17–29)	4 (2–4)	n/d
Jacket potato plain	8	13 (11–15)	16 (13–19)	15 (9–16)	18 (12–20)	1 (1–2) ^g^	1 (1–2) ^g^	n/d	n/d	22 (19–23)	29 (25–31)	0 (0–0.2) ^g^	n/d
Roast potatoes	5	13 (11–15) ^g^	16 (14–19) ^g^	10 (9–12)	12 (11–15)	10 (6–11)	12 (7–14)	n/d	n/d	17 (16–23)	22 (21–30)	2 (0.3–7)	n/d
*p*-value ^I^		0.011	0.011	>0.05	>0.05	<0.001	<0.001	n/a	n/a	>0.05	>0.05	0.003	n/a

* Data presented as median and interquartile (25th and 75th percentile); *n* = number of meals; n/d = no data available; n/a = not applicable; ^#^ 10 samples; ^~^ 39 samples; ^1^ significant difference between vegetarian and meat meal options (Mann–Whitney U test, *p* < 0.05); ^I^ significant difference within each meal category (Kruskal–Wallis). Significant difference between meal types within the same category (Mann–Whitney U test with Bonferroni adjustment, *p* < 0.05): ᵃ vegetarian lasagna, ᵇ macaroni and cheese, ^c^ fish and chips, ᵈ sausage and chips, ᵉ gammon and chips, ^f^ meat lasagna, ᵍ chips, ^h^ chili con carne and rice. Gammon = traditional British pork steak; chips = thick-cut French fries.
